# A new long-spined dinosaur from Patagonia sheds light on sauropod defense system

**DOI:** 10.1038/s41598-018-37943-3

**Published:** 2019-02-04

**Authors:** Pablo A. Gallina, Sebastián Apesteguía, Juan I. Canale, Alejandro Haluza

**Affiliations:** 1 Consejo Nacional de Investigaciones Científicas y Técnicas, Buenos Aires, Argentina; 2 Área de Paleontología, Fundación Félix de Azara, Universidad Maimónides, Hidalgo 775, C1405BCK Buenos Aires, Argentina; 3 Museo Municipal “Ernesto Bachmann”, Dr. Natali s/n, Villa El Chocón, 8311 Neuquén, Argentina

## Abstract

Dicraeosaurids are a group of sauropod dinosaurs characterized by a distinctive vertebral column with paired, long, neural spines, present in an extreme fashion in the South American form *Amargasaurus cazaui*. This distinctive morphology has been interpreted as a support structure for a thermoregulatory sail, a padded crest for display, a dorsal hump acting as fat reservoir, and even as inner cores for dorsal horns. Other inferred functions (if any) of this structure were related to sexual display and/or defense strategies. Here we describe a new dicraeosaurid sauropod, *Bajadasaurus pronuspinax* gen. et sp. nov., from Patagonia which preserves the most complete skull of the group and has extremely elongate bifid cervical neural spines that point permanently forward, irrespective of the neck position. Although much shorter versions of this neural spine configuration were already recorded for other dicraeosaurid taxa, the long, anteriorly bent spines of this new dinosaur support the hypothesis that these elongate spines of dicraeosaurid sauropods served as passive defense structures.

## Introduction

Since the finding of the nearly complete skeletons of *Dicraeosaurus* in the expeditions to the upper Jurassic “Saurian beds” of Tendaguru, Tanzania, led by Werner Janensch from the Geological-Paleontological Institute and Museum of the University of Berlin in the early 20th century (1909–1912), the presence of elongate bifid neural spines in the axial skeleton of this taxon was always a distinctive and iconic feature^1,2^. More than eighty years later, the discovery of a new, rather complete, dicraeosaurid skeleton from the Early Cretaceous of South America, *Amargasaurus cazaui*^3^, renewed discussions on the peculiar vertebral anatomy of these sauropod dinosaurs including interpretations as a support structure for a thermoregulatory sail, a padded crest as a display and/or clattering structure, a dorsal hump, or as internal cores of dorsal horns^4–6^.

At present, five other dicraeosaurid species are known from the Middle Jurassic of China (*Lingwulong shenqui*^7^), the Upper Jurassic of North America (*Suuwassea emiliae*^8^) and Central Patagonia (*Brachytrachelopan mesai*^9^), and the Early Cretaceous of North Patagonia (*Pilmatueia faundezi*^10^ and *Amargatitanis macni*^11,12^), but only *Brachytrachelopan* contributed to the knowledge of the entire cervical column, confirming the proposed shortening in the neck length within the group.

A new dicraeosaurid sauropod, *Bajadasaurus pronuspinax* gen. et sp. nov., from the Early Lower Cretaceous Bajada Colorada Formation (Northern Patagonia, Argentina), which includes dermal roof and palatal bones, a braincase, and a nearly complete lower jaw, expands the knowledge on the skull morphology of this group^13^. The preserved skull elements including the complete lower jaw allow the first reliable inference of the size and shape of a dicraeosaurid skull. Additionally, the skull was recovered in articulation with the anterior region of the neck and another cervical vertebra with exceptional development of anteriorly bent, bifid cervical neural spines, which informs hypotheses of defense behavior in sauropod dinosaurs. The temporal difference between *Bajadasaurus* and *Amargasaurus*, a 15 My younger spiny sauropod from the Neuquén basin, supports that the development of a fence of spines was likely adaptive over a long time period.

## Results

### Systematic palaeontology

SAUROPODA Marsh 1878

DIPLODOCOIDEA Marsh 1884

FLAGELLICAUDATA Harris & Dodson 2004

DICRAEOSAURIDAE Huene 1927

*Bajadasaurus pronuspinax gen.* et sp. nov.

### Etymology

Generic name from *Bajada* (Spanish for *downhill*, in reference to the locality Bajada Colorada) and *saurus* (Greek, lizard). Specific epithet from *pronus* (Latin, bent over forward) and *spinax* (Greek, spine), referring to the anteriorly pointed, curved, neural spines of the cervical vertebrae.

### Holotype

**Figure 1:**
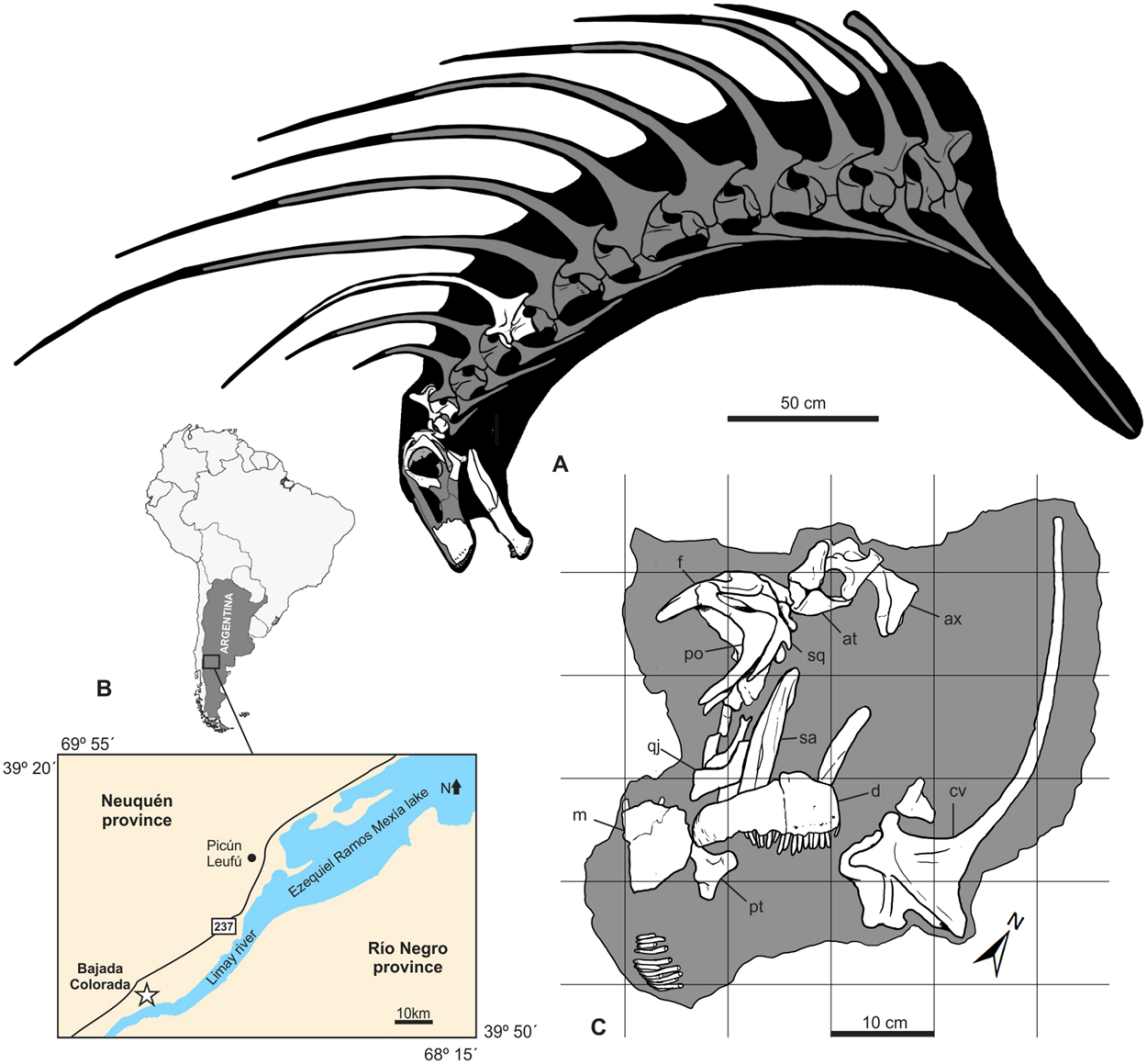
Skeletal reconstruction of *Bajadasaurus pronuspinax* gen. et sp. nov (MMCh-PV 75), location and quarry map. **(A**) The neck and skull reconstruction in left lateral view, showing preserved bones in white. The complete anterior cervical vertebra is located tentatively in the fifth position (see Description). The total count of cervical elements, as well as the relative extension of the neural spines, is based in the complete series of the related taxon *Amargasaurus*, the other dicraeosaurid with extremely elongated bifid neural spines along the neck. (**B**) A map of the surrounding area of the Ezequiel Ramos Mexía lake (Neuquén Province, Argentina) showing the type locality of *Bajadasaurus* (Bajada Colorada) indicated by a white star. (**C**) A quarry map showing the association and location of the remains in the field. at, atlas; ax, axis; cv, cervical vertebra; d, dentary; f, frontal; m, maxilla; po, postorbital; pt, pterygoid; qj, quadratojugal.

Museo Municipal Ernesto Bachmann (Villa El Chocón, Neuquén) MMCh-PV 75; a nearly complete skull (including left maxilla, left lacrimal, both prefrontals, both frontals, both parietals, both postorbitals, both squamosals, left quadratojugal, both pterygoids, both quadrates, supraoccipital, exoccipital-opisthotic complex, basioccipital, basisphenoid, both prootics, both laterosphenoids, both orbitosphenoids, both dentaries, left surangular, both angulars, both splenials, left prearticular, left articular, isolated upper tooth row), both proatlases, atlantal neurapophyses, axis and the ?fifth cervical vertebra (Fig. 1A).

### Locality and horizon

The remains were found in outcrops of the Lower Cretaceous (Late Berriasian–Valanginian^14,15^) Bajada Colorada Formation (Neuquén Basin, Patagonia, Argentina), at Bajada Colorada locality 40 km south of Picún Leufú town on the National Route 237 (Fig. 1B).

### Diagnosis

*Bajadasaurus pronuspinax* can be diagnosed by the following autapomorphies (marked by an asterisk), as well as a unique combination of character states: post-temporal fenestra extended medially with a long parietal contribution (*); basipterygoid processes extremely slender and long, more than six times longer than lateromedially wide (*); elongate angular, longer than the anteroposterior surangular length; neural spine of the axis oriented vertically (*); paired, anteriorly curved, and extremely elongate bifid neural spines of anterior-mid cervical vertebrae (*).

### Osteological description

Cranial bones and axial elements are preserved. The skull bones of *Bajadasaurus* include the dermal roof, braincase, palatal, and jaw elements. The axial remains consist of left and right proatlases, atlantal neurapophyses, axis and the ?fifth cervical vertebra (Figs 2 and S1–11).

**Figure 2:**
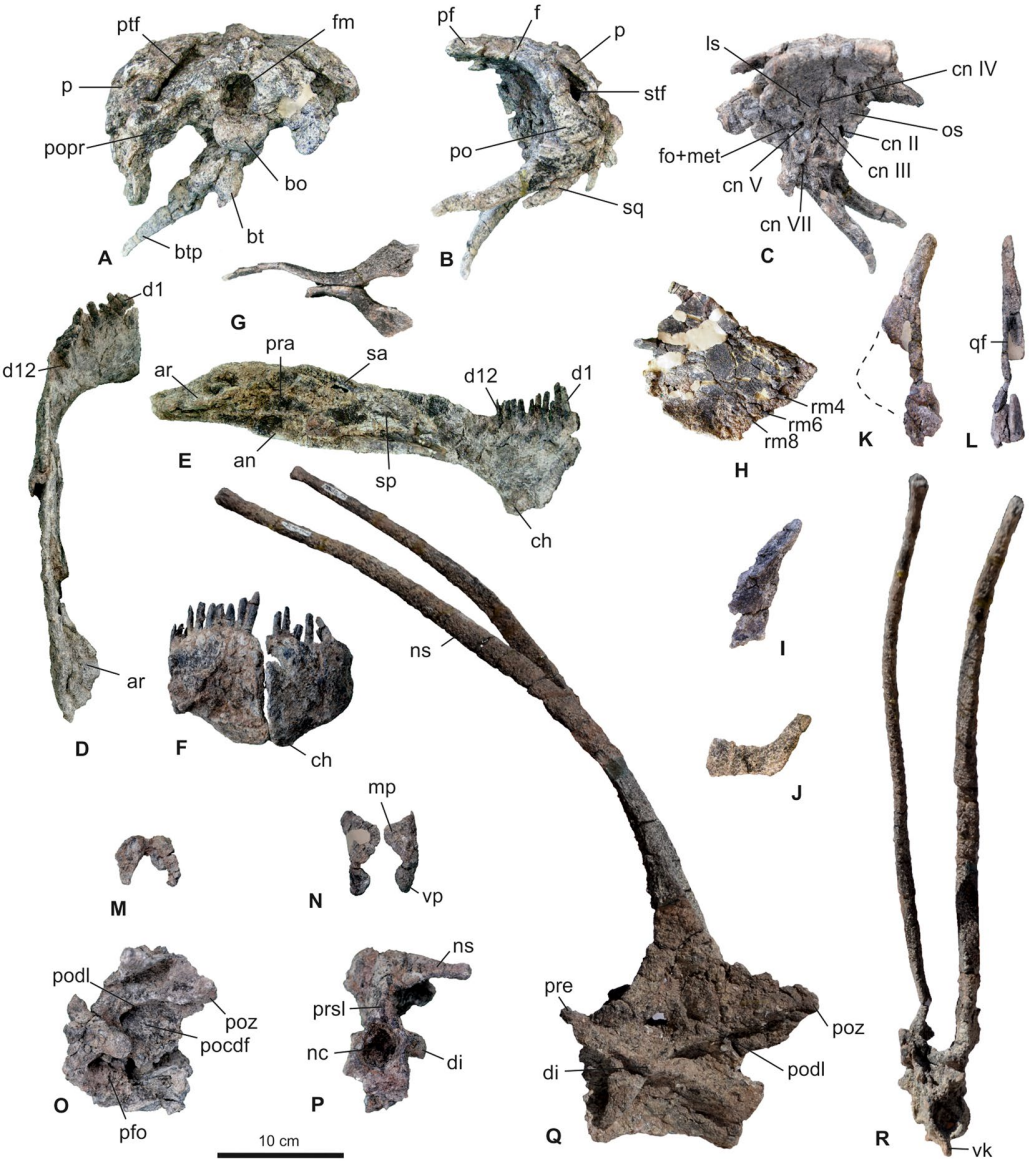
Skeletal elements of *Bajadasaurus pronuspinax* gen. et sp. nov (MMCh-PV 75). **(A**–**C**) Skull roof and braincase in posterior (**A**), left lateral (**B**) and right lateral (**C**) views. (**D**,**E**) Left lower jaw in dorsal (**D**) and medial (**E**) views. **F**, Dentaries in anterior view. (**G**) Pterygoids in ventral view. (**H**) Left maxilla in medial view. (**I**) Left lacrimal in lateral view. (**J**) Left quadratojugal in lateral view. (**K**,**L**) Right quadrate in medial (**K**) and posterior (**L**) views. (**M**) Proatlases in dorsal view. (**N**) Atlantal neurapophyses in anterior view. (**O**,**P)**, Axis in left lateral (**O**) and anterior (**P**) views. (**Q**,**R**) Fifth cervical vertebra in left lateral (**Q**) and anterior (**R**) views. an, angular; ar, articular; bo, basioccipital; bt, basal tubera; btp, basipterygoid process; ch, ‘chin’ of dentary; cn, cranial nerve; d, dentary; di, diapophysis; f, frontal; fm, foramen magnum; fo, fenestra ovalis; ls, laterosphenoid; met, metotic foramen; mp, medial process; nc, neural canal; ns, neural spine; os, orbitosphenoid; p, parietal; pfo, pneumatic fossa; po, postorbital; pocdf, postzygapophyseal centrodiapophyseal fossa; podl, postzygodiapophyseal lamina; popr, paraoccipital process; poz, postzygapophysis; pra, prearticular; pre, prezygapophysis; prsl, prespinal lamina; ptf, postemporal fenestra; qf, quadrate fossa; rm, replacement maxillary tooth; sa, surangular; sp, splenial; sq, squamosal; stf, supratemporal fenestra; vk, ventral keel; vp, ventral process.

The incomplete left maxilla is a flat and smooth bone that thickens in the dentigerous portion and thins toward the broken posterodorsal edge. The posterior ascending process is not preserved. However, the palatal shelf is visible in medial view as a thick horizontal lamina, as occurs in *Dicraeosaurus*^16^, *Diplodocus*^17^, and *Rapetosaurus*^18^. Eight alveoli are visible in medial view, with replacement teeth in most of them. This reduced count is only similar to *Suuwassea*^8^ (which possesses 7 alveoli, although the maxilla may be incompletely preserved) within diplodocoids which commonly bear more than 10–12 teeth in the maxilla (10–11 in *Diplodocus*^19^; 12 in *Kaatedocus*^20^; 12 in *Dicraeosaurus*^16^; 24 in *Nigersaurus*^21^).

The left lacrimal is nearly complete. The dorsal half is triangular in section with a straight lateral ridge as in *Dicraeosaurus*^16^, and unlike the projecting spur seen in *Kaatedocus*^22^. The ventral portion is anteroposteriorly broad and flat. A small foramen is present on the orbital face, unlike the broad foramen recognized in *Dicraeosaurus*^16^. The jugal contact seems to be stepped as in *Diplodocus* sp. (CM 11161).

Both prefrontals are wide-based triangular bones in dorsal view. A small participation of the prefrontal in the dorsal orbital rim is present, unlike the bigger contribution seen in *Dicraeosaurus*^16^ and *Amargasaurus* (MACN-N 15) in which the prefrontals are also proportionally larger and more robust.

The frontals are strongly fused to the parietals, which dorsally cover them in the mid-posterior region. In dorsal view, the frontals are broad posteriorly and narrow anteriorly, with a markedly sigmoidal lateral border, differing from the less pronounced border present in *Diplodocus* (CM 11161), *Amargasaurus* (MACN-N 15), and *Dicraeosaurus*^16^. As a result, the orbits are dorsally exposed. A similar condition is present in *Lingwulong*^7^. Both frontoparietal and postparietal foraminae are taphonomically joined along the skull midline, as a consequence of the broken bony bridge between them. The small frontoparietal foramen resembles the condition present in *Suuwassea*^8^, differing from the larger foramina seen in *Amargasaurus* (MACN-N 15) and *Dicraeosaurus*^16^. The frontals have a small contribution to the anterior margin of the supratemporal fenestra.

The parietals are anteroposteriorly extended over the skull roof. They are curved posteriorly with anterior crescent-shaped crests as in *Amargasaurus* (MACN-N 15). However, the muscular depressions anteriorly bounded by these crests are shallow as in *Dicraeosaurus*^16^ and *Suuwassea*^8^, differing from the deep depression seen in *Amargasaurus* (MACN-N 15). The postparietal foramen reaches the border of the supraoccipital crest. The supratemporal fenestra is roofed by the posterior segment of the parietal.

The postorbital has two main processes which contact the frontal dorsally and the jugal anteroventrally. While the orbital border is rounded, the posterior one is marked by a straight angle with a reduced indistinct posterior process, as in *Amargasaurus* (MACN-N 15) and *Dicraeosaurus*^16^.

The squamosal is massive posteriorly and becomes slender anteriorly, forming an enlarged anterior squamosal process, which strongly suggests a contact with the quadratojugal, differing from Diplodocidae. This contact ventrally frames a narrow lateral temporal fenestra that extends posterodorsally. Additionally, the medioventral surface of the squamosal is concave for the articulation with the quadrate head. In the posteroventral corner, a prominent, ventrally directed ‘prong’ is developed, as commonly occurs in dicraeosaurids^23^.

The left quadratojugal is a flat, transversely compressed bone, which ventrally borders a narrow lateral temporal fenestra. It is formed by two rami oriented at an obtuse angle, a situation only present in diplodocids (e.g., *Diplodocus* sp. CM 11161) and titanosaurs (e.g., *Tapuiasaurus*^24^). The maximum dorsoventral height of the anterior ramus is twice the minimum dorsoventral height.

Both pterygoids are partially preserved. They are complex, nearly planar, bow-like bones with four main processes. Two of them point posteriorly, enclosing an embayment that meets the pterygoid wing of the quadrate. The other two processes point anteriorly. The anterodorsal process is flat and short. The anteroventral process is long and narrow. A dorsoventral constriction occurs at one third of the total length, where a smooth crest develops to contact the long basipterygoid processes (Fig. S5).

Both quadrates are partially preserved. They are triradiate bones, with an elongate posterodorsal process, a short, rounded and low anterior pterygoid wing, and a ventral condyle. The shaft is posteriorly concave. A shallow fossa is present at mid-length on the medial surface, as occurs in *Suuwassea*^8^ and diplodocids^22^. In posterior view, a shallow longitudinal fossa is present. Ventrally, the articular condyle has a roughly triangular shape as in *Suuwassea*^8^ and diplodocids^23^.

The supraoccipital and exoccipital-opisthotic complex are completely fused. The supraoccipital is rhomboidal and bears a distinct and narrow sagittal nuchal crest as in other dicraeosaurids and *Kaatedocus*^20^. However the dorsal margins are nearly straight, unlike the stepped borders recognized in *Dicraeosaurus*^16^, *Amargasaurus* (MACN-N 15), and *Suuwassea*^8^. The post-temporal fenestra extends medially in *Bajadasaurus*, which represents an autapomorphy. The exit for cranial nerve XII in the dorsolateral corner of the occipital condyle is not discernable due to poor preservation. The anteroposteriorly flat paraoccipital processes project posteroventrally. The crista tuberalis is not robust but clearly delimits a shallow fossa in which the fenestra ovalis and the metotic foramen open posteriorly. A conspicuous crista prootica with a lateral expansion at its ventral end is present, as is common in dicraeosaurids^22,25^.

The basioccipital forms the main body of the occipital condyle and the paired basal tubera. Unlike *Amargasaurus* (MACN-N 15) and *Dicraeosaurus*^16^, the articular surface of the condyle does not exceed the width of the condylar neck. The condyle is dorsoventrally compressed.

The basal tubera hang from the base of the condylar neck and extend ventrally with a slightly convex posterior surface. A vertical sulcus extends along the midline of the tubercles. The basal tubera are slightly narrower than the occipital condyle as in rebbachisaurids and other dicraeosaurids^23^.

The basisphenoid forms the gracile and elongated basipterygoid processes, which extend anteroventrally. Unlike *Dicraeosaurus*^16^ and *Amargasaurus* (MACN-N 15), where these processes are robust structures and somewhat shorter, in *Bajadasaurus* they are longer and extremely slender (more than six times longer than lateromedially wide), thus representing a possible autapomorphy of the taxon. The diverging angle is narrow, less than 30 degrees, as in other Dicraeosauridae. The external foramen for the internal carotid artery opens on the lateral surface, where the basipterygoid processes and the basal tubera meet, as occurs in *Amargasaurus*^26^.

The prootic is a triangular and flat plate of bone with a reduced laterally exposed surface. Although no sutures are visible, the crista antotica and the opening for cranial nerve V may represent the limits with the laterosphenoid, as in other sauropods^26^. The opening for cranial nerve VII is located at the base of the crista prootica as in *Amargasaurus* (MACN-N 15), just above the external foramen for the internal carotid artery.

The laterosphenoid is completely fused with the prootic posteriorly and the orbitosphenoid anteriorly. The contact with the frontal is quite evident by the presence of an anteroposteriorly oriented suture, as occurs in *Amargasaurus* (MACN-N 15). The crista antotica is short, posterodorsally oriented, and disappears at the level of the opening for cranial nerve V, contrasting with the more conspicuous and extended crista antotica present in *Amargasaurus* (MACN-N 15) and *Dicraeosaurus*^16^. The foramina for cranial nerves II, III and V are horizontally aligned with respect to the skull roof.

Both subtriangular orbitosphenoids are plate-like bones that meet at the sagittal plane anteriorly. Paired optic foramina for cranial nerve II open at mid height, unlike *Suuwassea*^8^ and *Kaatedocus*^20^ in which the openings remain unpaired. Anterodorsally, the orbitosphenoids frame the exit of the cranial nerve I, which is partially compressed.

The dentary shows a symphyseal segment (oblique to the sagittal plane), a curved segment (corner), and the base of the posterior rami. Differing from the more squared jaws of diplodocids and rebbachisaurids^17,21^, it is a J-shaped element, as in other dicraeosaurids. It is a slender bone as in *Suuwassea*^27^, unlike the thick dentary of *Dicraeosaurus*^16^. Only the anterior region and the corner are dentigerous. It bears twelve full-grown teeth, some of which have unworn distal tips. The anteroventral margin of the dentary shows a dorsoventrally deep ‘chin’ as in flagellicaudatans^28,29^, and a labial prominence near the symphysis as in *Dicraeosaurus*^16^ and *Suuwassea*^27^. The subtriangular symphysis tapers ventrally as in *Dicraeosaurus*^16^ and *Suuwassea*^27^. The anterior external surface bears small, ovoid foramina. Medially, a posterior triangular embayment develops in which the surangular and the angular overlap. A shallow coronoid eminence is recognized behind the tooth row as is common in diplodocoids^30^.

The surangular is a transversely flat and elongated bone. It maintains constant depth and straightness until the articular region, where it curves ventrally and tapers. The retroarticular process is short relative to most diplodocids^31^. A small surangular foramen is anterodorsally located in the lateral surface. In medial view the surangular is partially covered by the articular and prearticular posteriorly, and the splenial anteriorly.

The angular is extremely elongated, and longer than the surangular, unlike the condition seen in diplodocids. The posterior region tapers dorsoventrally and curves slightly ventrally. Ventrally, the angular is straight except for the retroarticular segment, which curves medially.

The posterior half of the splenial is flat and covers the anterior portion of the surangular and a small portion of the angular medially. The posteroventral process is tongue-like, unlike *Diplodocus*^17^ where it tapers distally. This morphology is convergently present in titanosaurs such as *Nemegtosaurus*^32^ and *Tapuiasaurus*^24^.

The prearticular is a thin plate of bone, vertically located, that tapers posteriorly. Although incomplete, the anterior portion indicates the presence of a narrow adductor fossa unlike the conspicuous one seen in *Diplodocus*^17^. Posteriorly, an oblique flat process extends medially on the retroarticular region.

The wedge-shaped articular is tightly located between the surangular laterally, the prearticular medially and the angular ventrally. Dorsally, the articular shows a triangular perimeter with two main surfaces divided by a low ridge (perpendicular to the sagittal plane), located behind the glenoid region.

Twenty-four upper teeth in anatomical position were found in close association with the left maxilla. This count equals the tooth row of the dentary. Both upper and dentary teeth are narrow-crowned (Slenderness Index: 4.6), peg-like elements. They are nearly straight or slightly curved medially. Some of them show extremely reduced, low angled, single planar wear facets.

Left and right proatlases were preserved in articulation with the skull. They are fin-like triangular bones with an ovoid broad base, flat sides, and pointed distal ends as in *Kaatedocus*^20^ and *Dicraeosaurus*^2^.

The atlantal neurapophyses are triangular, wing-like thin bones, laterally convex and medially concave. The posterodorsal projection is rounded distally as in *Amargasaurus*^26^ and *Galeamopus*^33^, unlike the distally tapering ones present in *Kaatedocus*^20^. A medial rounded process projects anterodorsally at mid-length of the neurapophyses, as in *Amargasaurus*^26^ and *Suuwassea*^34^.

The axis is nearly complete, although the neural spine is broken and displaced laterally. The total height is twice the total length of the centrum, as in *Dicraeosaurus*^2^. The axial centrum is twice as long as posteriorly tall, as in other dicraeosaurids. The body is laterally constricted at mid-length. Large undivided pneumatic fossae are present as in *Dicraeosaurus*^2^, *Suuwassea*^8^ and *Amargasaurus* (MACN-N 15). The tall neural arch rests on the entire centrum. Deep triangular postzygapophyseal centrodiapophyseal fossae are present, as in *Dicraeosaurus*^2^ and *Amargasaurus* (MACN-N 15). The small diapophysis points backwards as in *Suuwassea*, unlike the ventrally pointed diapophyses of *Dicraeosaurus*^2^ and *Amargasaurus* (MACN-N 15). In anatomical position, the narrow vertical neural spine is non-bifurcated, triangular in cross-section and tapers distally, differing from other sauropods. The postzygapophyses and the spinopostzygapophyseal laminae embrace a deep triangular spinopostzygapophyseal fossa.

The ?fifth cervical vertebra is the most characteristic element of *Bajadasaurus*. With an extremely elongate, bifurcated neural spine, this vertebra is four times taller than long, only comparable with *Amargasaurus* (MACN-N 15). However, *Bajadasaurus* possesses anteriorly curved and slightly laterally pointed neural spines that differ from any other known sauropod. The centrum is two times longer than posteriorly tall and shallow undivided fossae are located along the lateral sides, as in *Dicraeosaurus*^2^, *Amargasaurus* (MACN-N 15), and *Pilmatueia*^10^ (MLL-Pv-004). However, *Pilmatueia* (MLL-Pv-004) differs from *Bajadasaurus* in having three small foramina and a deep centrodiapophyseal fossa associated to the lateral excavation of the centrum. The centrodiapophyseal fossa is absent in *Bajadasaurus*. Ventrally, the centrum of *Bajadasaurus* narrows in a longitudinal keel unlike other dicraeosaurids such as *Pilmatueia*^10^, *Dicraeosaurus*^2^ and *Brachytrachelopan*^9^, which develop a ventral keel in a transversely wide concave surface. These, plus other differences between *Pilmatueia*^10^ and *Bajadasaurus* such as the absence of median tubercle between the elongated neural spines in the latter and the phylogenetic position of both contemporaneous dicraeosaurid sauropods (see Phylogenetic analysis bellow) justify the taxonomic separation of both taxa. The neural arch is nearly as long as the centrum length with the neural spine base being located at the midpoint. This condition, along with the general proportions and laminar and/or apophyses (zygapophyses and diapophyses) arrangements are comparable with the fifth cervical of *Dicraeosaurus*^2^, the ?sixth of *Brachytrachelopan*^9^ and the seventh of *Amargasaurus* (MACN-N 15). In this context we tentatively assign this cervical vertebra to the fifth position. Stout epipophyses are located above the postzygapophyses. The rod-like, 58 cm long neural spines, maintain an ovoid section along their length except for the transversely compressed triangular base. A peculiar trait is that the tip of the spine is not acute as in *Amargasaurus* (MACN-N 15), but slightly expanded. The poor preservation of bone surfaces in the specimen precludes the recognition of longitudinal striations on the spine surface as those observed in *Amargasaurus*^6^, and inferred to support an external keratinized horn sheath. However, considering the extremely elongation of the neural spine (a condition very similar to that of *Amargasaurus*), external horn sheaths could be ascribed to *Bajadasurus* as well (see Discussion).

### Phylogenetic analysis

**Figure 3:**
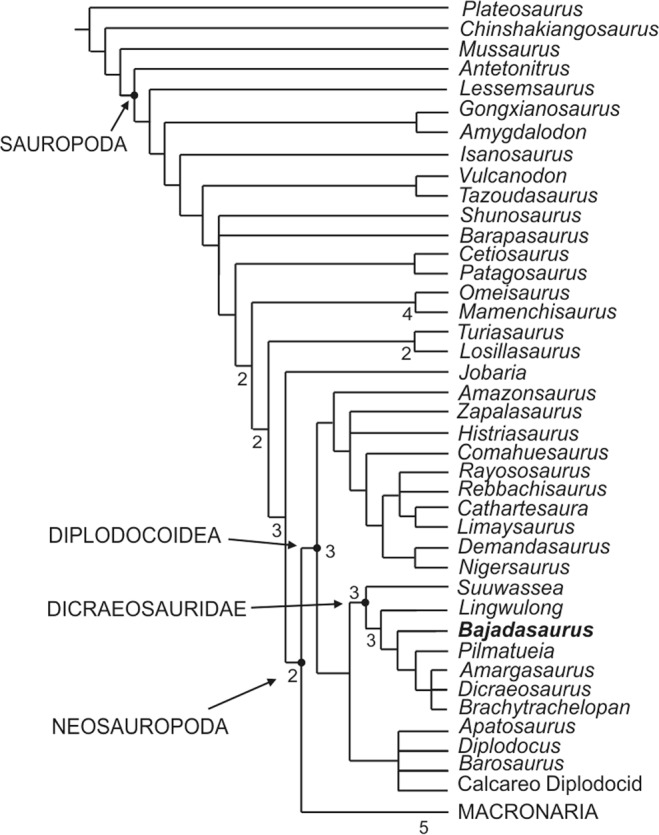
Phylogenetic position of *Bajadasaurus pronuspinax* gen. et sp. nov (MMCh-PV 75) within Dicraeosauridae (see Supplementary Information). Bremer support values higher than one are shown.

The phylogenetic affinities of *Bajadasaurus* were evaluated in the context of a previous data set^7^ (see Methods and Supplementary Information for details). The analysis retrieved 820 most parsimonious trees of a length of 1114 steps. The strict consensus tree shows a large polytomy at the base of Neosauropoda, which can be resolved if two unstable taxa (*Amargatitanis macni*^11,12^ and *Erketu ellisoni*^35^) are pruned from the MPTs (see Supplementary Information). A reduced strict consensus tree recovered *Bajadasaurus* well nested within the family Dicraeosauridae (Fig. 3, Supplementary Fig. 12), sharing six synapomorphies with all dicraeosaurids, plus five synapomorphies shared with *Lingwulong*, *Pilmatueia*, *Brachytrachelopan*, *Dicraeosaurus* and *Amargasaurus*. *Bajadasaurus* is recovered as the sister taxon of a clade consisting of the lower Cretaceous *Pilmatueia* plus an unresolved derived group including the Cretaceous *Amargasaurus*, the Jurassic *Brachytrachelopan*, and *Dicraeosaurus*, supported by the presence of a supratemporal fenestra with a maximum diameter subequal to that of the foramen magnum (char. 47), and basipterygoid processes with an angle of divergence less than 30° (char. 69).

## Discussion

**Figure 4:**
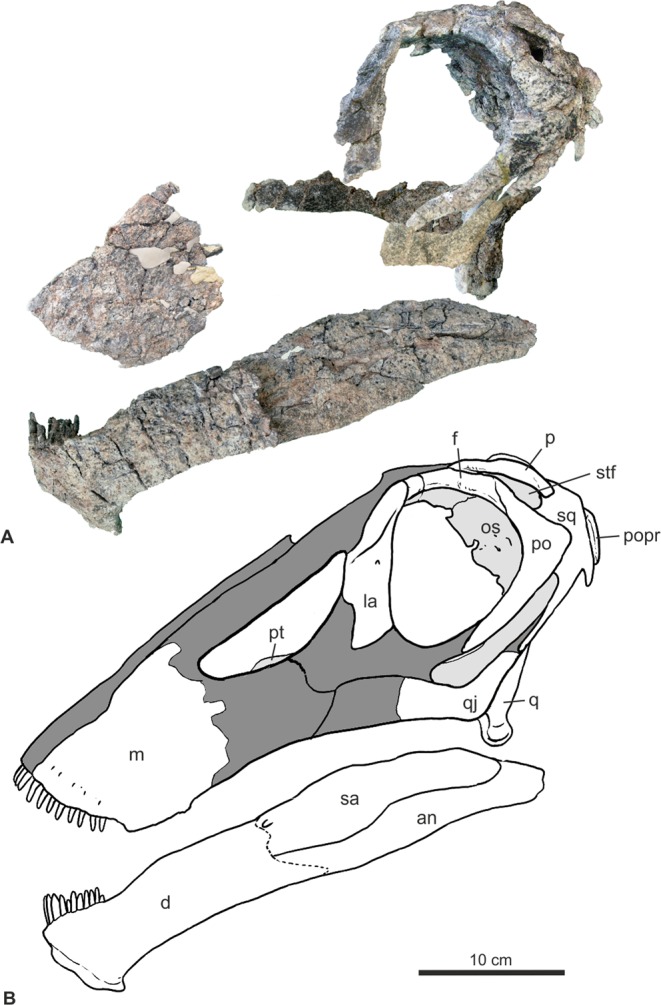
Skull of *Bajadasaurus pronuspinax* gen. et sp. nov (MMCh-PV 75). (**A**) Cranial bones of *Bajadasaurus* in articulation in lateral view. The spatial location of disarticulated elements is inferred based on recognizable articular facets and/or by comparison with the skull of *Diplodocus* sp. (CM 11161). (**B**) Interpretative drawing of the skull of *Bajadasaurus* in lateral view. Missing bones are reconstructed based on the skull of *Diplodocus* sp. (CM 11161). an, angular; d, dentary; f, frontal; la, lacrimal; m, maxilla, os, orbitosphenoid; p, parietal; po, postorbital; popr, paraoccipital process; pt, pterygoid; q, quadrate; qj, quadratojugal; sa, surangular; sq, squamosal; stf, supratemporal fenestra.

The cranial bones of *Bajadasaurus* show several novelties in the skull of dicraeosaurid sauropods. As shown in Fig. 4, this gracile skull with dorsally exposed orbits, dorsoventrally compressed occipital condyle, extremely narrow basipterygoid processes, elongate and slender anterior processes of the squamosals, medially extended post-temporal fenestrae, short lateral temporal fenestrae and a reduced dentition in the maxilla and dentary, largely differs from other known taxa within Dicraeosauridae (Fig. 4). Additionally, the probable contact between the squamosal and quadratojugal, an anteroposteriorly extended and gracile lower jaw, and a narrow, posteriorly extended lateral temporal fenestra are recognized for the first time in a dicraeosaurid skull. The dorsally exposed orbits mean, in the context of a head-down normal position for the *Amargasaurus* skull^26^ that eyes were capable of a forward-directed, perhaps stereoscopic view while feeding.

The most striking feature of *Bajadasaurus* is the presence of extremely long cervical neural spines that curve anteriorly. Axial skeletons with deeply bifid neural spines are common in dicraeosaurid sauropods. In fact, *Amargasaurus cazaui* shows practically the same development of cervical neural spine elongation as *Bajadasaurus*, but the spines of the former point backwards rather than forwards. On the contrary, *Dicraeosaurus* and *Brachytrachelopan* show anteriorly inclined neural spines in the cervical vertebrae, although the spines are much shorter than in *Bajadasaurus*.

The discovery of *Amargasaurus cazaui* opened a field of research and assumptions concerning the function of its spines. Different tetrapods acted as models and the extracorporeal exposition of the main portion of the spines of *Amargasaurus* was compared to that of iguanodontians as support of a thermoregulatory sail^36^, a dorsal hump^5^, a padded crest related to sexual selection^37^, or an internal core for dorsal horns^6^. All but the last explanation require that these long and extremely gracile bone projections, now recognized in *Bajadasaurus* as well, can support enough physical stress to avoid fracturing, based on similar studies in mammals^38,39^. Conversely, the presence of external keratinous horns in the distal-most two-thirds of the dorsal extension of the neural spines provides a better mechanical solution against a potential fracture.

A recent publication described the supravertebral pneumatic diverticles of *Amargasaurus* as occupying the ventral 1/3 of the mid**-**cervical vertebrae, and in anterior and posterior cervicals, the ventral half and the space between the metapophyses^6^. The remaining 2/3 of the distal spine was recognized as having an undulated and striated surface, comparable to bones bearing keratinized sheath-covered structures. This is also supported by the absence of a supraspinous ligament attachment on the top of neural spines in *Bajadasaurus* and *Amargasaurus*, thus differing from the related *Dicraeosaurus*^6^.

Although bone is stronger and stiffer in passive situations depending on strain and load, horns and other keratin-based materials are tougher and highly resistant to impact-related fractures in dry to mesic conditions both in mammalian^40,41^ or sauropsid keratin^42^. Thus, the cover sheath may transfer the load to the bony core along a radial direction. Additionally, resistance is improved in slightly bent horns^39^, and in sheaths much longer (double, as in some artiodactyls) than neural spines, thus reducing impulsive loads more than other geometries. This physical characteristic would be biologically beneficial considering that this portion of the neural arch (including its base) is particularly vulnerable because it acts as the osseous roof of the neural cord. Hence, a possible breakage in life could result in a dangerous and traumatic damage for the animal, with serious immunological trauma. Breakages of vertebral laminae in humans (laminectomy) revealed that trauma arises when fragments displace into the spinal canal^43^. Breakages in *Dimetrodon* sails revealed a fast-healing system by osteoclastic resorption and deposition of lamellar bone^44^.

In most extant sauropsids, except for the rostral horn of *Trioceros jacksoni*, and lateral spines of gerrhosaurid lizards, sheaths do not reach 100% of the bony core length. The discovery of an exceptionally preserved specimen of the ankylosaur *Borealopelta* permitted examination in extinct archosaurs^45^. In ankylosaurs, the keratinous sheath of the parascapular spine extends to 25% up the bony core length. Although previous authors considered that horns were no longer than the bone itself in *Amargasaurus*^6^, the length ratios of bone core/keratinous sheath in reptiles and mammals and ongoing research on material resistance suggest that the keratinous sheath in *Amargasaurus* and perhaps *Bajadasaurus* should have been more than 50% longer than the bone core to improve the bone protection^46^.

While the extension of the neural spines may be speculative for *Bajadasaurus*, the bone core itself goes much beyond the anterior limit of the head, becoming a front fence for the body. In this context, whereas the ?fifth vertebra points to a low position respect to a grazing head, we could hypothesize that following neck vertebrae with larger centra and higher reach should logically be longer. A defensive explanation based only on neural spine osseous resistance seems to be incompatible with the 15 My persistence of the *Bajadasaurus-Amargasaurus* long-spined dicraeosaurid strategy. The vast group of acute spines with long protective sheaths would represent a disturbing fence for a loitering carnivore. Under moderate charge, the breakage would affect only spine tips, preserving the bone core within them.

## Methods

### Phylogenetic analysis

The phylogenetic position of *Bajadasaurus pronuspinax* was tested through an equally weighted parsimony analysis in TNT v.1.1^47^. The data matrix used was based on a previously published phylogeny which included a wide array of sauropodomorph taxa^7^, with the addition of *Amargatitanis macni*^11,12^, *Pilmatueia faundezi*^10^, and *Bajadasaurus pronuspinax*. This dataset included 375 characters and 76 taxa (Supplementary Information). The dataset was analyzed starting from 5000 replicates of Wagner trees followed by TBR branch swapping and saving 10 tress per replicate, reaching the best score 1060 times. The trees recovered were subjected to an additional round of branch swapping. Unstable taxa were identified search by Pruned Tree command in TNT. Bremer support values^48^ were calculated to evaluate the robustness of the nodes in the reduced strict consensus tree.

### Institutional abbreviations

CM, Carnegie Museum of Natural History, Pittsburgh, PA, United States; MACN, Museo Argentino de Ciencias Naturales “Bernardino Rivadavia”, Buenos Aires, Argentina; MLL-Pv, Museo Municipal de Las Lajas, Vertebrate Paleontology, Las Lajas, Neuquén, Argentina.

## Supplementary information

Supplementary Information
