# Supplementary material for: A new long-spined dinosaur from Patagonia sheds light on sauropod defense system
Source: Sci Rep. 2019 Feb 4;9:1392. doi: 10.1038/s41598-018-37943-3 (PMC6362061; doi:10.1038/s41598-018-37943-3)
Supplement: Supplementary file 1 — Supplementary Information [file 41598_2018_37943_MOESM1_ESM.pdf]

A new long-spined dinosaur from Patagonia sheds light on sauropod defense system

Pablo A. Gallina, Sebastián Apesteguía, Juan I. Canale and Alejandro Haluza

Supplementary Information

1. Characters and Taxa
2. Phylogenetic assumption
3. Phylogenetic results
4. Synapomorphies
5. Supplementary Figures
6. Table of measurements
7. Supplementary References

1. Characters and Taxa

The character list include in this analysis is the same of Xu et al.,[1], as well as the taxa include with the addition of *Amargatitanis macni*[2,3] , *Pilmatueia faundezi*[4] and *Bajadasaurus pronuspinax.*

2. Phylogenetic assumption

The following characters were treated as ordered (actual character number): 12, 58, 95, 96, 102, 106, 108, 115, 116, 119, 120; 145, 152, 163, 213, 216, 232, 233, 234, 235, 252, 256, 298, 299, and 301.

3. Phylogenetic results

The analysis retrieved 820 most parsimonious trees of length 1114 steps. The strict consensus shows a great politomy at the base of Neosauropoda. In order to recognize unstable taxa we compared pruned trees, and resulted number of nodes gained, using the Pruned Tree command in TNT. *Erketu ellisoni* was recovered in multiple positions such as a diplodocid, a basal macronarian, a basal titanosauriform, and both basal and derived titanosaurian. *Amargatitanis macni* was recovered in different position within Flagellicaudata (Supplementary Fig. 12). As a consequence, a reduced strict consensus tree was generated via a posteriori deletion of both unstable taxa.

4. Synapomorphies supporting *Bajadasaurus* within Diplodocoidea, Flagellicaudata, Dicraeosauridae and *Lingwulong*+more derived Dicraeosauridae (actual character number)

Diplodocoidea

Char. 145 1→2

Char. 163 1→0

Char. 184 0→1

Char. 205 0→1

Char. 215 0→1

Flagellicaudata

Char. 8 0→1

Char. 12 1→2

Char. 53 1→0

Char. 82 0→1

Char. 86 0→1

Char. 132 0→1

Char. 158 0→1

Char. 193 0→1

Char. 198 0→1

Char. 216 1→2

Char. 286 0→1

Char. 295 1→0

Char. 327 0→1

Char. 370 0→1

Dicraeosauridae

Char. 4 0→1

Char. 26 0→1

Char. 34 1→0

Char. 50 0→1

Char. 85 0→1

Char. 371 0→1

Lingwulong+more derived Dicraeosauridae

Char. 35 0→1

Char. 113 1→0

Char. 135 1→0

Char. 139 1→0

Char. 374 0→1

5. Supplementary Figures

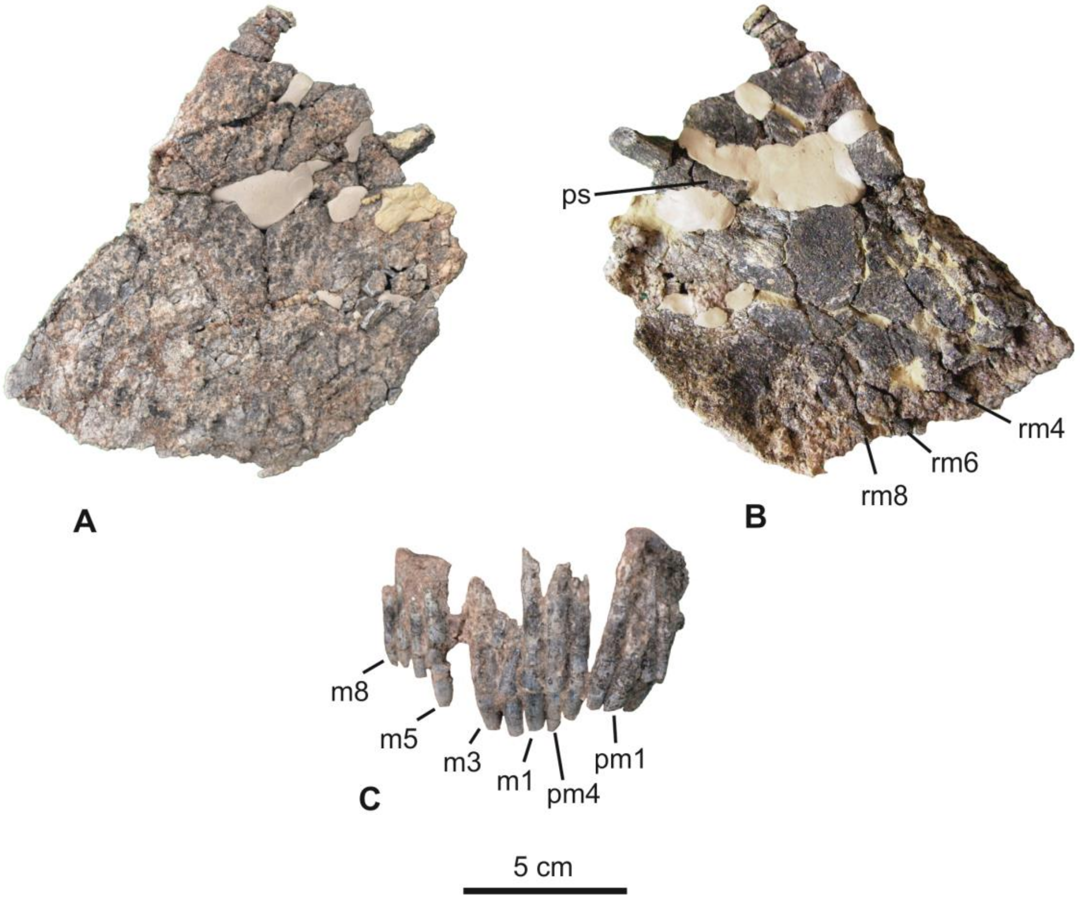

Supplementary Figure 1. Left Maxilla and upper teeth of *Bajadasaurus pronuspinax* gen. et sp. nov. (MMCh-PV 75). Maxilla in lateral (A) and medial (B) views. Associated upper teeth in lateral (C) view. m, maxillary tooth; pm, premaxillar tooth; ps, palatal shelf; rm, replacement maxillary tooth.

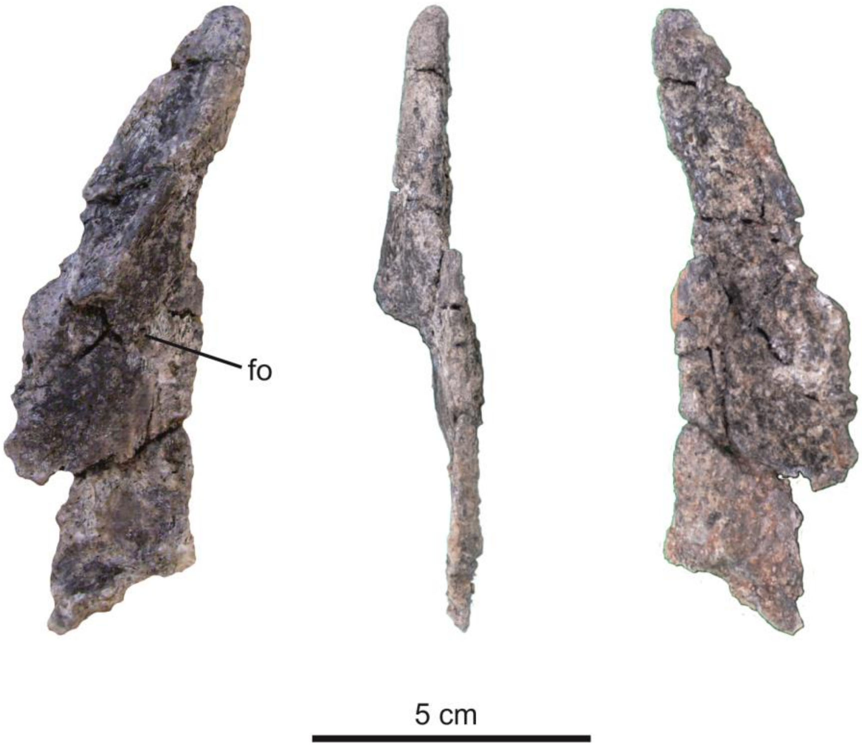

Supplementary Figure 2. Lacrimal of *Bajadasaurus pronuspinax* gen. et sp. nov. (MMCh-PV 75). Anterior (A) and medial (B) views. fo, foramen.

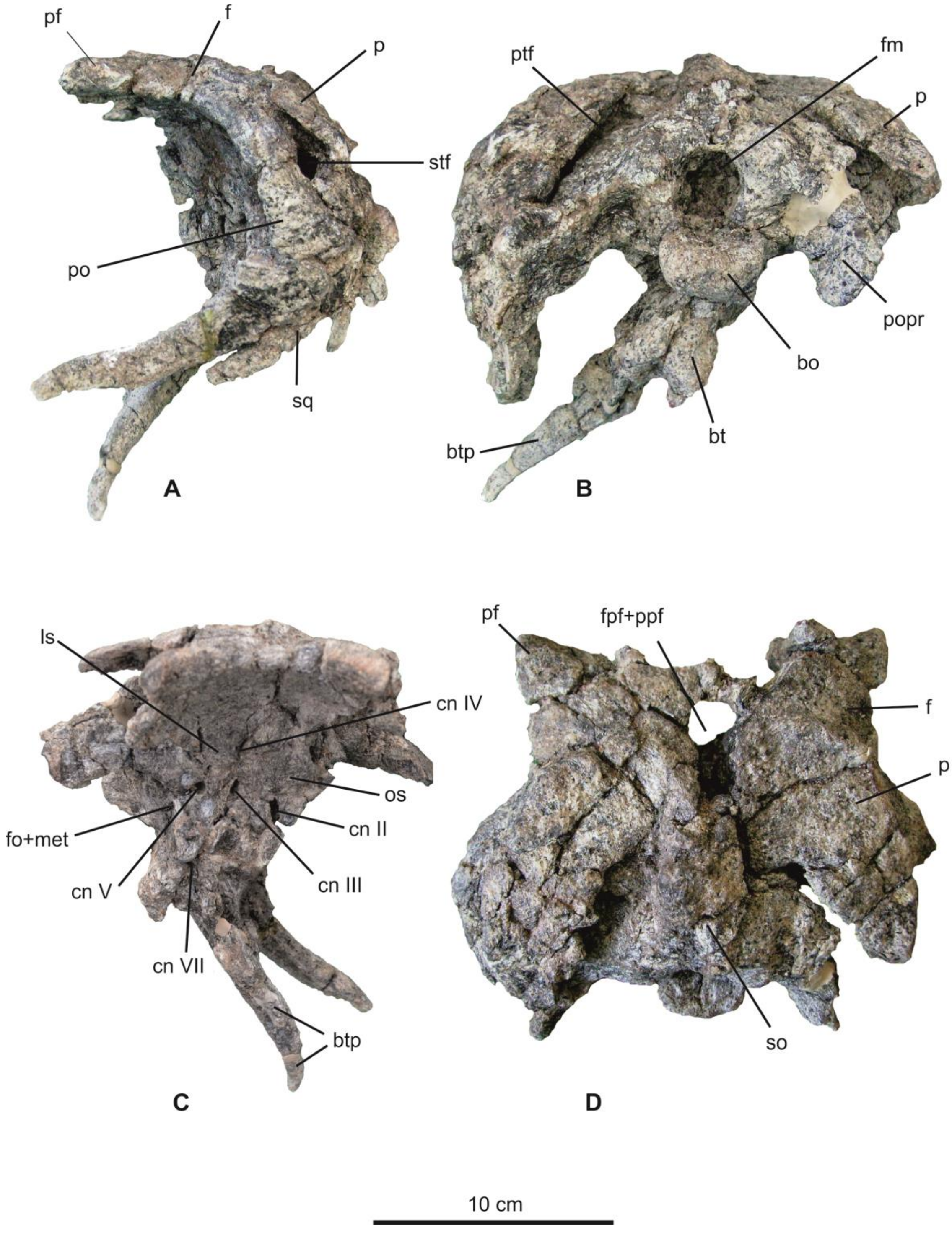

Supplementary Figure 3. Skull roof and braincase of *Bajadasaurus pronuspinax* gen. et sp. nov. (MMCh-PV 75). Left lateral (A), posterior (B), right lateral (C), and dorsal (D) views. Abbreviations as in Figure 2.

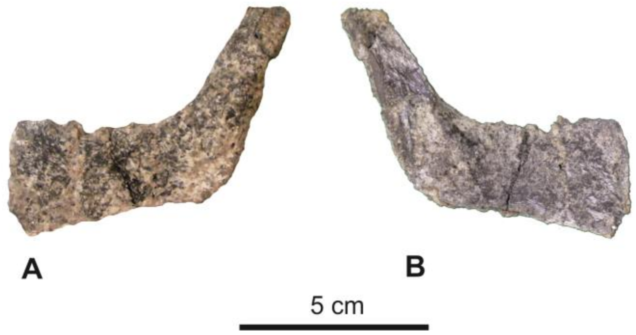

Supplementary Figure 4. Quadratojugal of *Bajadasaurus pronuspinax* gen. et sp. nov. (MMCh-PV 75). Lateral (A) and medial (B) views.

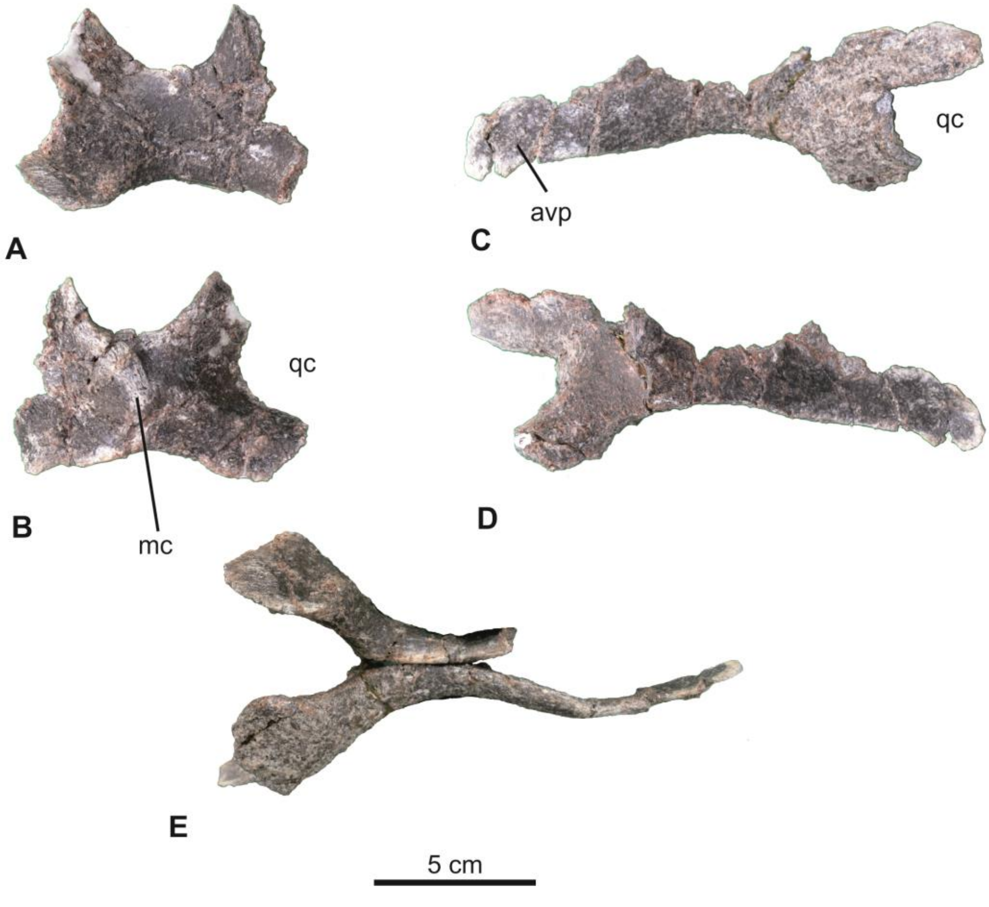

Supplementary Figure 5. Pterygoids of *Bajadasaurus pronuspinax* gen. et sp. nov. (MMCh-PV 75). Right pterygoid in lateral (A) and medial (B) views. Left pterygoid in lateral (C) and medial (D) views. Both pterygoids in ventral (E) view (anterior towards right). avp, anteroventral process; mc, medial crest; qc, contact for the quadrate.

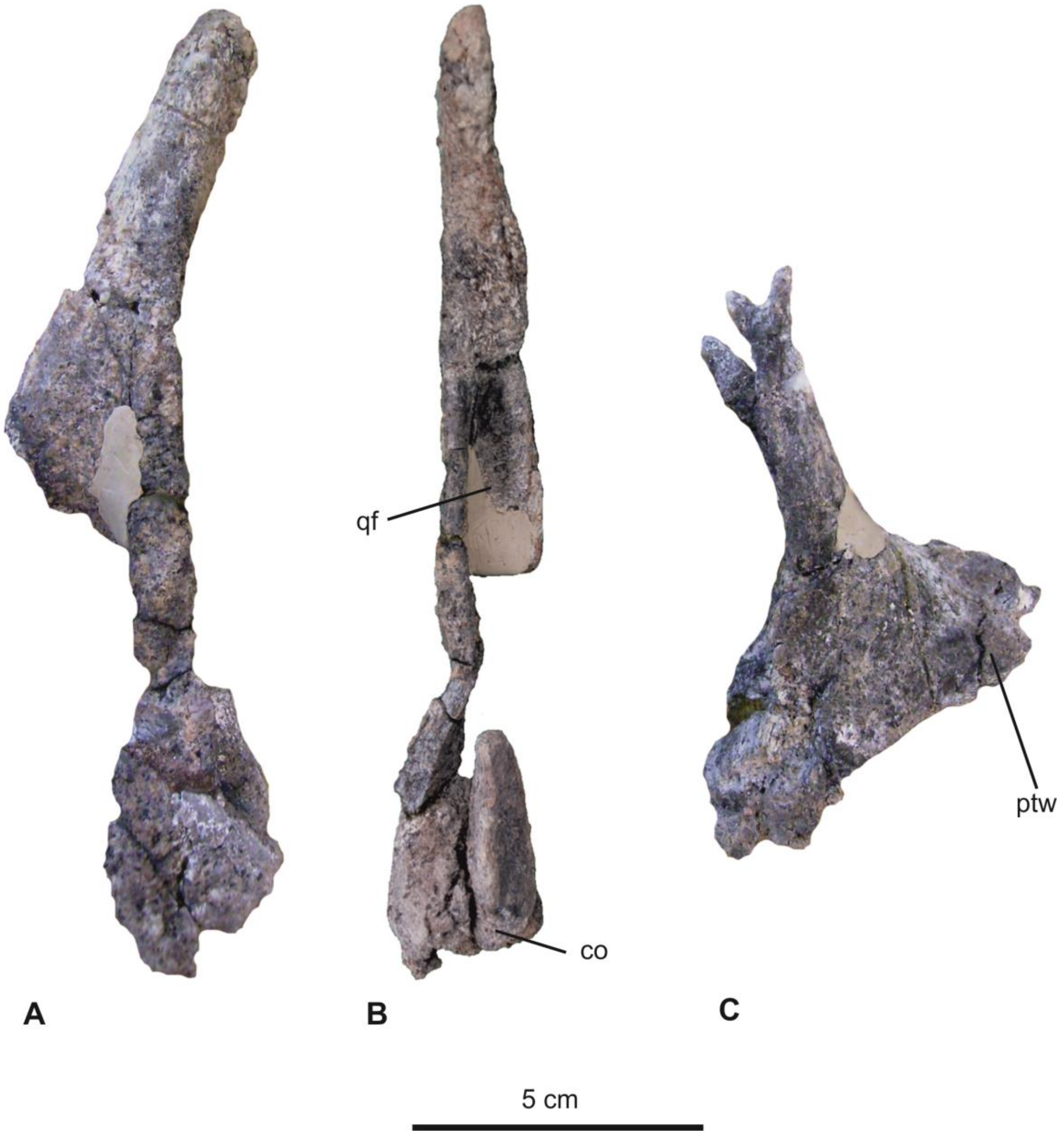

Supplementary Figure 6. Quadrates of *Bajadasaurus pronuspinax* gen. et sp. nov. (MMCh-PV 75). Right quadrate in medial (A) and posterior (B) views. Left quadrate in medial (C) view. co, condyle; ptw, pterygoid wing; qf, quadrate fossa.

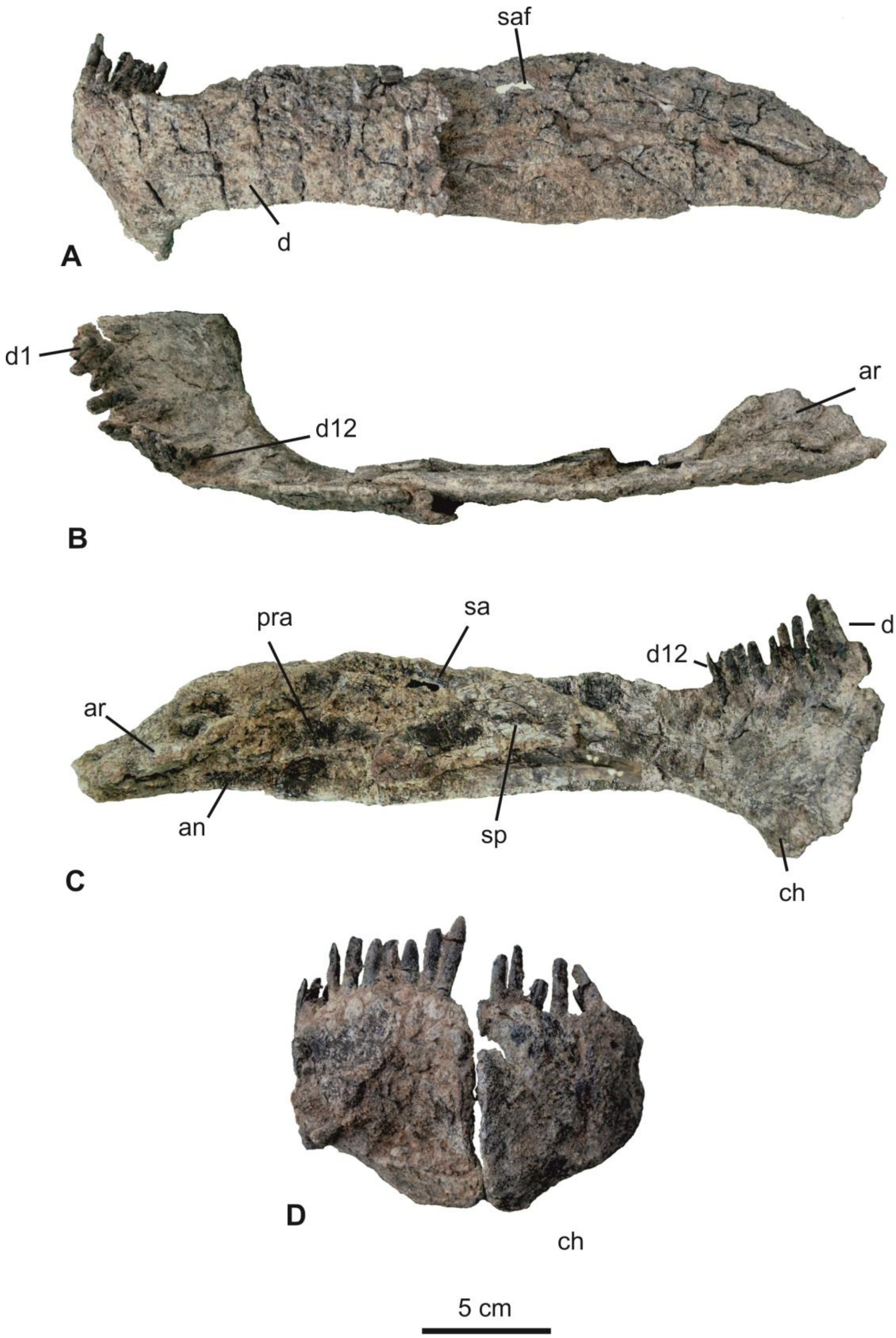

Supplementary Figure 7. Lower jaw of *Bajadasaurus pronuspinax* gen. et sp. nov. (MMCh-PV 75). Left lower jaw in lateral (A), dorsal (B), and medial (B) views. Both dentaries' in anterior (D) view. Abbreviations as in Figure 2.

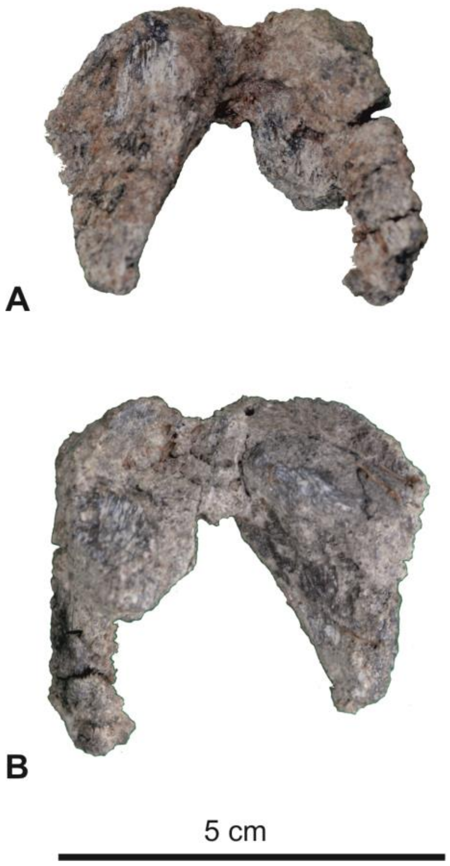

Supplementary Figure 8. Proatlas of *Bajadasaurus pronuspinax* gen. et sp. nov. (MMCh-PV 75). Dorsal (A) and ventral (B) views.

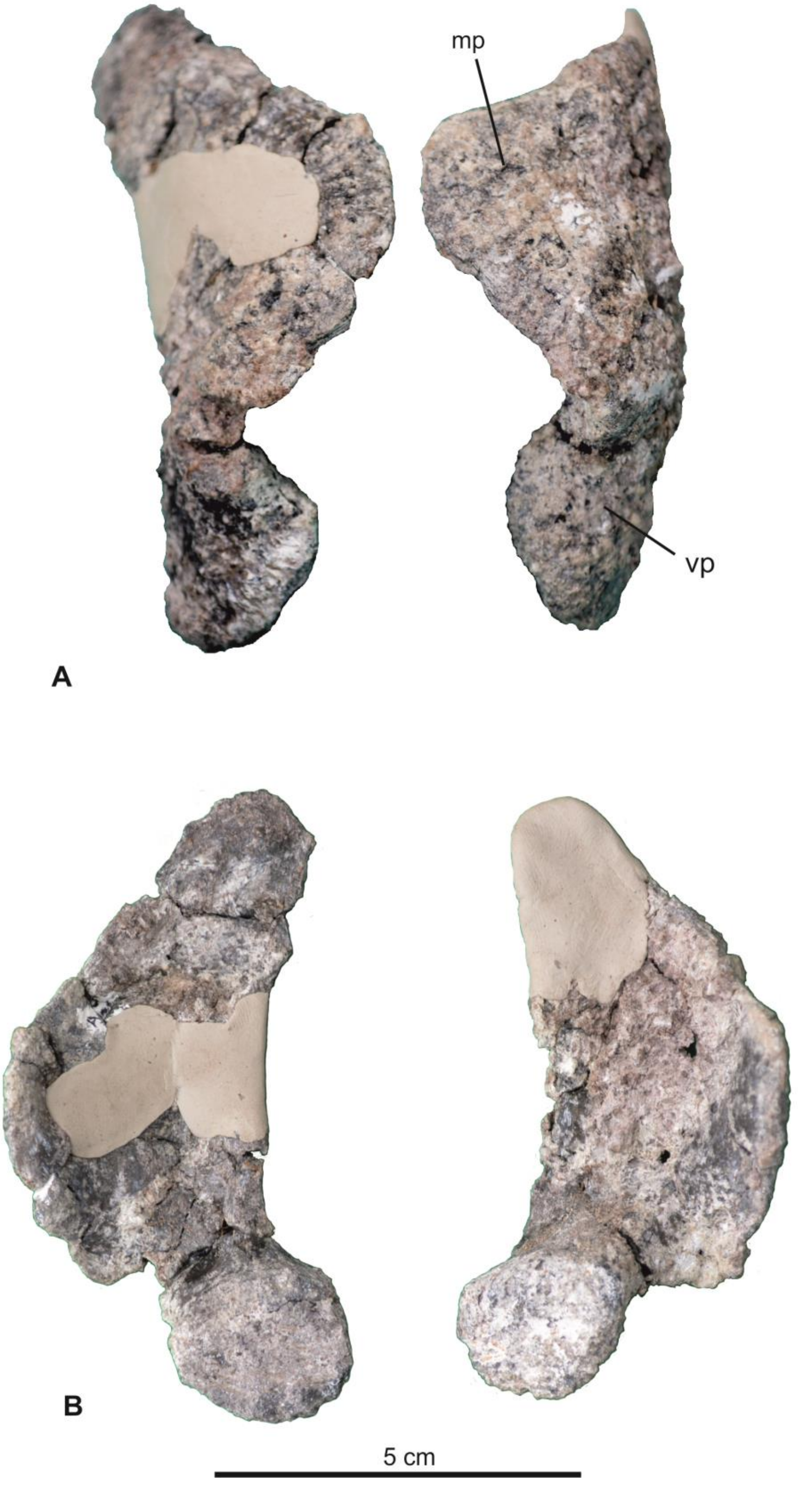

Supplementary Figure 9. Atlantal neurapophyses of *Bajadasaurus pronuspinax* gen. et sp. nov. (MMCh-PV 75). Anterior (A) and medial (B) views. Abbreviations in Figure 2.

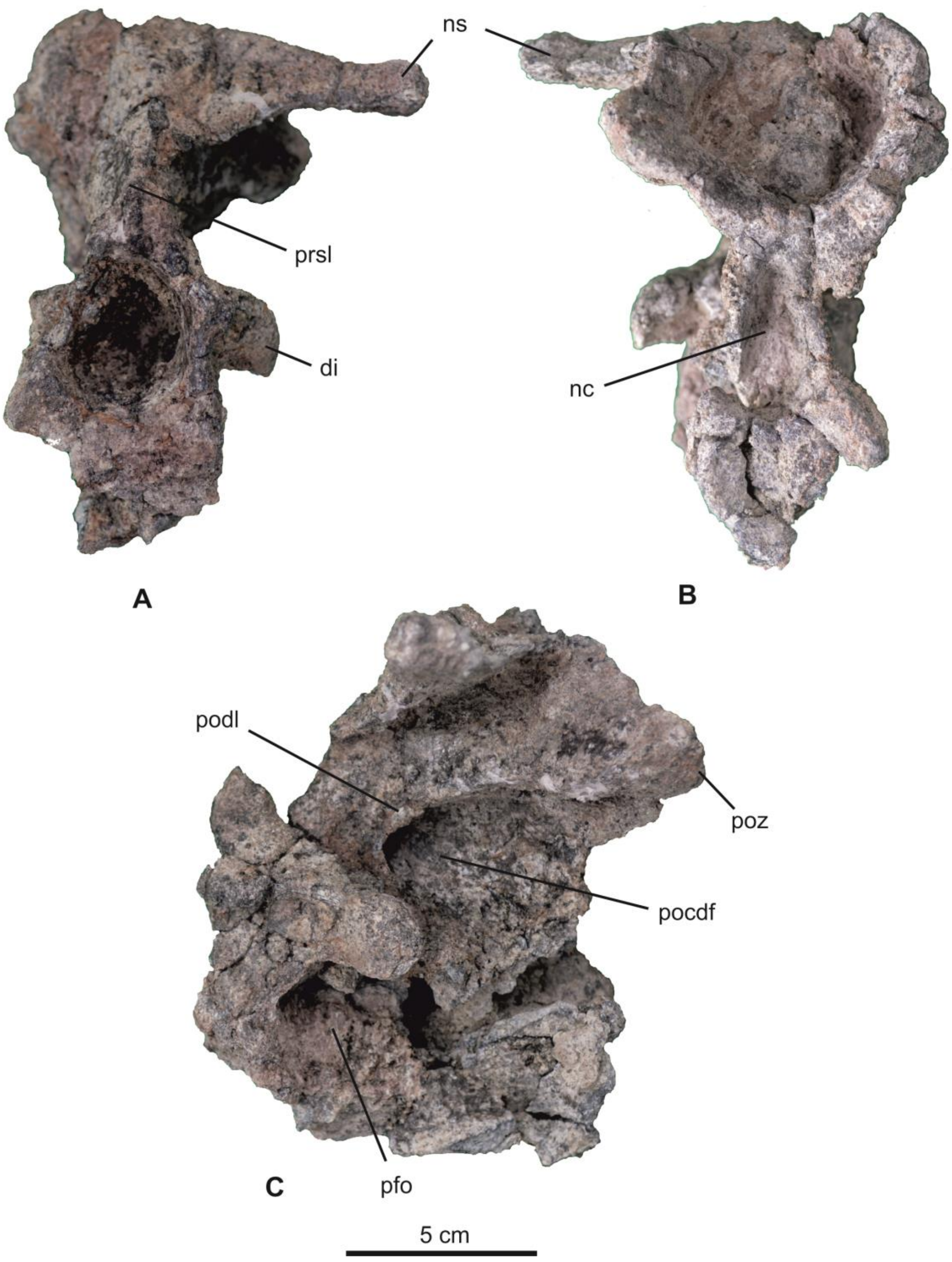

Supplementary Figure 10. Axis of *Bajadasaurus pronuspinax* gen. et sp. nov. (MMCh-PV 75). Anterior (A), posterior (B), and lateral (C) views. Abbreviations as in Figure 2.

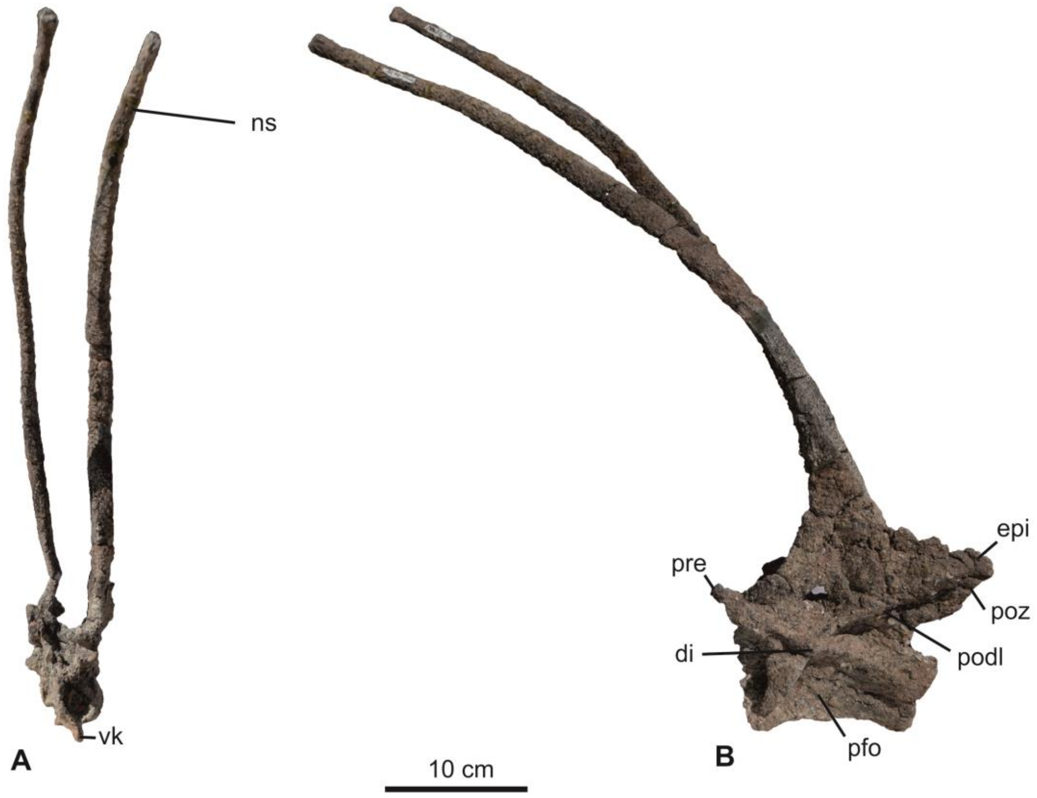

Supplementary Figure 11. ?Fifth cervical vertebra of *Bajadasaurus pronuspinax* gen. et sp. nov. (MMCh-PV 75). Posterior (A) and lateral (B) views. Abbreviations as in Figure 2.

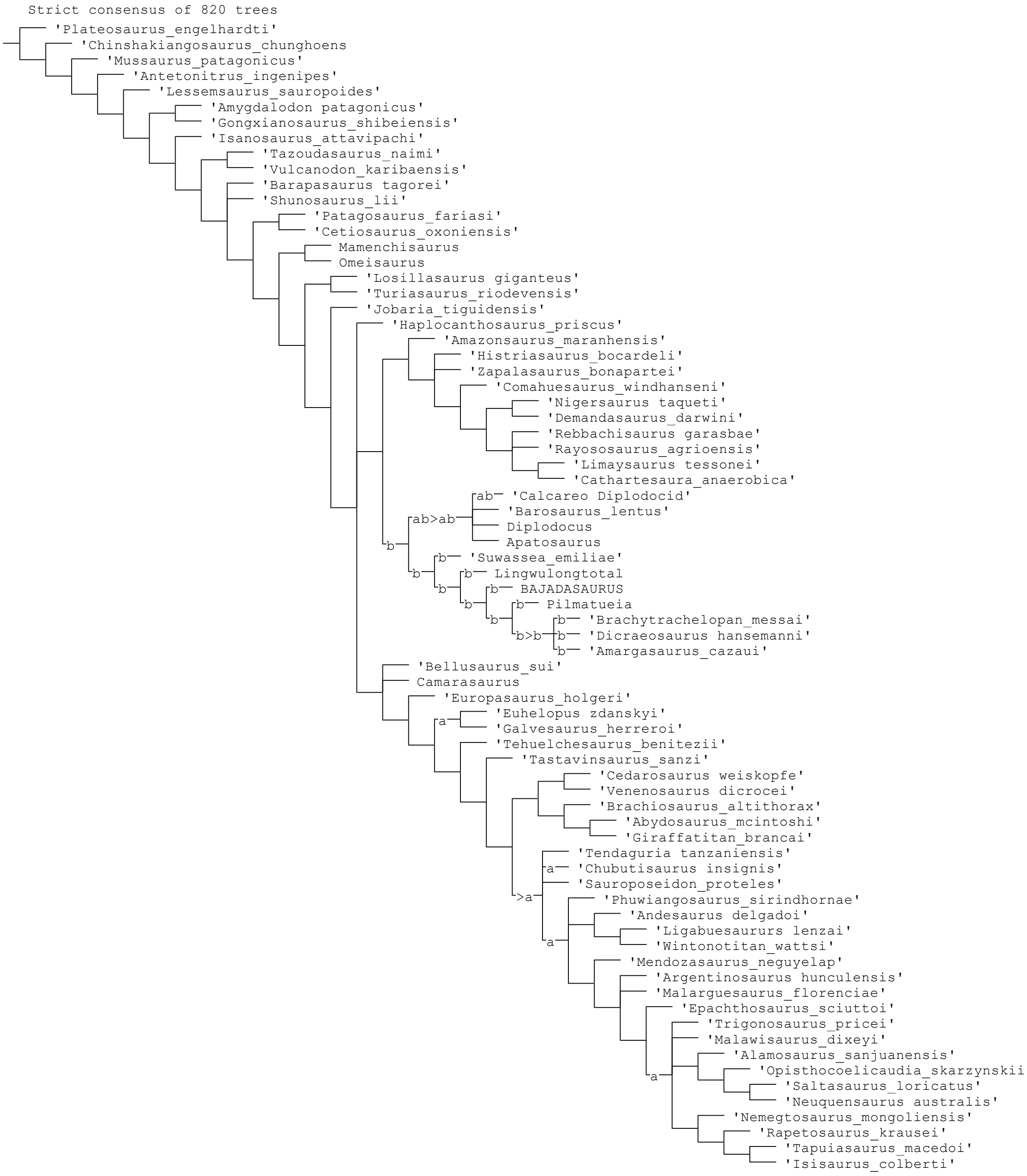

Supplementary Figure 12. Reduced strict consensus tree showing the unstable positions of *Erketu ellisoni* (a) and *Amargatitanis macni* (b).

6. Skull and vertebral bones measurements of *Bajadasaurus pronuspinax* gen. et sp. nov. (MMCh-PV 75) (in mm, > indicates estimated measurement)

Element	side	apL	tbL	dvH	minW	maxW	lmL	hrdvH	vrapW	aaW	aaH	paW	paH	apnaL	naW	naH	sH
Maxilla	L	132	63	>127	2	18											
Prefrontal	L	57					62										
	R	53					63										
Frontal	L	>79					76										
	R	87					55										
Postorbital	L	60		128	12	34		26	26								
	R	64		124	11	28		28	24								
Quadratojugal	L	>60		>52	2	10		24	14								
Lacrimal	L	30		113	2	14											
Quadrate	L	53				25											
	R			163		25											
Squamosal	L	63		>92		23											
	R	>53		134		22											
Parietal	L	33					47										
	R	31					46										
Supraoccipital				34													
Paroccipital process (exoccipital-opisthotic)	L			27			74										
	R			28			>51										
Occipital condyle				16		32											
Foramen magnum				28		19											
Basal tubera	L			36		14											
	R			36		16											
Basipterygoid process	L			110													
	R			>74													
Pterygoid	L	153		49			50										
	R	>88		46			62										
Dentary	L	144		73			69										
	R	113		73			63										
Surangular	L	208		27													
Angular	L	205		29													
Proatlas	L	32		36		15											
	R	28		32		18											
Atlantal neurapophysis	L	>66		49													
	R	83		63													
Axis		93								46	49	45	40	107	78	75	66
5th? cervical vertebrae		126								_	67	47	63	210	72	59	61

Abbreviations

aaH: anterior articular surface height; **aaW:** anterior articular surface width; **apL:** anteroposterior length; **apnaL:** anteroposterior length of neural arch; **dvH:** Dorsoventral heigth; **hrdvH:** horizontal ramus dorsoventral height; **lmL:** lateromedial length; **maxW:** maximum transverse width; **minW:** minimum transverse width; **naH:** neural arch heigth; **naW:** neural arch width; **paH:** posterior articular surface height; **paW:** posterior articular surface width; **sH:** spine height; **tbL:** length tooth-bearing portion; **vL:** length ventral edge; **vrapW:** ventral ramus anteroposterior width.

Measurements protocols

Maxilla
apL: measured with the maxilla oriented as in figure 4
dvH: measured with the maxilla oriented as in figure 4

Postorbital
apL: corresponds to the length of the dorsal horizontal ramus
dvH: corresponds to the length of the ventral ramus
hrdvH: measured at mid length
vrapW: measured at mid length

Quadrate
apL: measured at the level of the pterygoid wing

Basal tubera
dvH: measured from the ventral edge of the occipital condyle

Basipterygoid process
dvH: measured from the ventral edge of the basal tubera

Dentary
dvH: measured at symphysis
lmL: measured along the anterior, toothed, sector of dentary

Proatlas
apL: measured at the base

Cervical vertebrae
apL: anteroposterior length of centrum
naH: measured from the neurocentral suture to the base of the neural spine
naW: measured between postzygapophyses

7. Supplementary References

1. Xu, X., Upchurch, P., Mannion, P. D., Barrett, P. M., Regalado-Fernandez, O. M., Mo, J., Ma, J. & Liu, H. A new Middle Jurassic diplodocoid suggests an earlier dispersal and diversification of sauropod dinosaurs *Nat. Comm.* **9**, 2700 (2018)
2. Apesteguía, S. The sauropod diversity of the La Amarga Formation (Barremian), Neuquén (Argentina). *Gondwana Res*. **12**, 533–546 (2007).
3. Gallina, P. A. Reappraisal of the Early Cretaceous sauropod dinosaur *Amargatitanis macni* (Apesteguía, 2007), from northwestern Patagonia, Argentina. *Cretaceous Res.* **64**, 79–87 (2016).
4. Coria, R. A., Windholz, G. J., Ortega, F. & Currie, P. J. A new dicraeosaurid sauropod from the Lower Cretaceous (Mulichinco Formation, Valanginian, Neuquén Basin) of Argentina. *Cret. Res.* **93**, 33–48 (2019).
